# Intravitreal Bevacizumab as Anti-Vascular Endothelial Growth Factor in the Management of Complications of Proliferative Diabetic Retinopathy

**Published:** 2013

**Authors:** J. Fernando Arevalo

**Affiliations:** Retina Division, Wilmer Eye Institute, Johns Hopkins University School of Medicine, Baltimore, USA & The Vitreoretinal Division, King Khaled Eye Specialist Hospital, Riyadh, Kingdom of Saudi Arabia & Faculty of Health Sciences, Stellenbosch University, South Africa

**Keywords:** Bevacizumab, Anti-Vascular Endothelial Growth Factor, Diabetic Retinopathy

## Abstract

Vascular endothelial growth factor (VEGF) has been shown to be an endothelial cell-specific mitogen and an angiogenic inducer in a variety of in vitro and in vivo models. Furthermore, it has been demonstrated to increase retinal vessel permeability by increasing the phosphorylation of tight junction proteins. Recent work has found elevated levels of VEGF in ocular fluids of patients with proliferative diabetic retinopathy (PDR). Thus, it makes sense to consider anti-VEGF treatments in the management of PDR. The purpose of the current research is to determine if intravitreal bevacizumab as anti-VEGF is helpful in the management of complications of PDR.

## INTRODUCTION

Dietary habits and an increase in sedentarism are the main culprits of a worldwide epidemic in diabetes mellitus (DM). In the year 2010, more than 285 million people suffered from DM. The World Health Organization has estimated that by the year 2030 there will be 370 million persons affected with DM in the world, and every one of them will be at risk of developing diabetic retinopathy (DR) [1]. Other studies predict that the number will be even higher at 439 million persons affected with DM in the world [2]. Prior to the advent of panretinal photocoagulation (PRP), proliferative diabetic retinopathy (PDR) was the main cause of diabetic blindness. 

More than 60 years ago, Michaelson speculated on the presence of a Factor X that was capable of inducing retinal angiogenesis or neovascularization [3]. Vascular endothelial growth factor (VEGF) appears to be the most likely candidate to be Michaelson’s factor X and the main molecular mediator in diabetic retinopathy. The underlying problem in diabetic patients is the progressive retinal ischemia caused by the metabolic disarray of chronic hyperglycemia. Hypoxia is a major inducer of VEGF gene transcription. VEGF has been shown to be an endothelial cell-specific mitogen and an angiogenic inducer in a variety of in vitro and in vivo models [4]. Furthermore, it has been demonstrated to increase retinal vessel permeability by increasing the phosphorylation of tight junction proteins. Recent work has found elevated levels of VEGF in ocular fluids of patients with PDR [5-10]. Thus it makes sense to consider anti-VEGF treatments in the management of diabetic macular edema (DME) and PDR. 

Several anti-VEGF agents are currently available in clinical practice. Pegaptanib sodium (Macugen®, Eyetech, NY, USA) is an aptamer against the VEGF-165 isoform [11]. Ranibizumab (Lucentis®, Genentech, San Francisco, CA, USA) is a fragment of a humanized monoclonal antibody against all VEGF isoforms [12]. Bevacizumab (Avastin®, Genentech, San Francisco, CA, USA) is a humanized, recombinant monoclonal IgG antibody that binds and inhibits all VEGF isoforms [13-15]. Aflibercept, previously known as VEGF-Trap eye (Eylea®, Regeneron Pharmaceutics Inc., Tarrytown, NY) is a recombinant fusion protein that consists of portions of human VEGF receptors 1 and 2 that allows it to bind to VEGF-A, VEGF-B and placental growth factor [16]. All of these agents have been used to differing extents in the management of DME and PDR [17-27]. However, there is a tremendous difference in price between these drugs. Bevacizumab costs only a small fraction of Ranibizumab, Pegaptanib or Aflibercept. If Bevacizumab were not available and research for its off-label use not performed, many diabetics in developing countries would not have enjoyed the benefit of this remarkable medication.

We have long been interested in studying the effects of intravitreal bevacizumab in several vitreoretinal conditions including DME and PDR [20-27]. Given the off-label nature of intravitreal bevacizumab, its pharmacokinetics and safety have not been as thoroughly studied as other approved similar drugs. We have previously shown in an open label uncontrolled clinical study that intravitreal bevacizumab appears to be safe and well tolerated [28]. A recent randomized clinical trial has shown no differences with regards to systemic side effects between intravitreal ranibizumab and intravitreal bevacizumab [29]. 

## HYPOTHESIS

Intravitreal bevacizumab (IVB) may decrease retinal neovascularization in patients with PDR after six months of follow-up. However, the effect may decrease at 24 months of follow up due to tachyphylaxis, and pan-retinal photocoagulation and/or vitrectomy will be necessary. 

## DISCUSSION

The underlying problem in diabetic retinopathy is progressive retinal ischemia caused by the metabolic disarray of chronic hyperglycemia. If the hyperglycemia is not controlled, up-regulation of growth factors and cytokines occur. VEGF is an important, if not the most important cytokine involved. Activation of the VEGF-receptor pathway triggers a network of signaling processes that promotes endothelial cell growth, migration, survival from pre-existing vessels, differentiation and mobilization of endothelial progenitor cells from the bone marrow into the peripheral circulation. An increase in vascular permeability followed by angiogenesis leads to retinal, optic disc and even anterior segment neovascularization [30]. 

PRP has been the mainstay of treatment for PDR, and its suppressive effect on ocular neovascularization has been well documented [31]. However, substantial regression of new vessels may take weeks after completion of PRP, and in up to one-third of cases, new vessels continue to grow despite initial PRP [32]. In these cases, vitreous hemorrhage may induce visual loss and prevent the need for complete laser. Moreover, macular edema may increase after PRP and cause transient or persistent visual loss [33]. Neovascularization on and around the optic disc (NVD) and vitreous hemorrhage were found to be more frequently associated with severe visual loss despite PRP in the Diabetic Retinopathy Study (DRS) and ETDRS [34-35]. Long intervals between PRP sessions and the variable amount of time required for a favorable response may increase the incidence of complications due to the progression of PDR [35]. In fact, a single episode of PRP or shorter intervals between PRP episodes, although desirable in severe PDR, and when the patient must travel long distances for treatment, are often associated with acute visual disturbances due to exudative choroidal detachment, retinal detachment, and macular edema [32,36-38]. Several reports document the regression of retinal neovascularization following the administration of intravitreal anti-VEGF compounds in PDR [39-41]. 

We previously conducted a retrospective study in 43 eyes of 39 patients with PDR that had persistent retinal neovascularization and concomittant DME [24]. Of the total of 43 eyes, 31 (72.1%) eyes had been previously treated with scatter photocoagulation at least six months before intravitreal bevacizumab, no eyes had prior focal/ grid laser photocoagulation, and no eyes had a previous intravitreal triamcinolone injection. Twenty-four (55.8%) eyes were treated with an intravitreal injection of 1.25 mg and 19 (44.2%) eyes were treated with 2.5 mg of bevacizumab. 

At the end of the two year follow-up (Restrepo et al. The Association for Research in Vision and Ophthalmology Annual Meeting 2010. Fort Lauderdale, FL. Unpublished data), the mean baseline BCVA of logMAR = 0.94 ± 0.38 ETDRS (20/176) improved to a mean BCVA of logMAR = 0.67 ± 0.39 ETDRS (20/94) (p < 0.0005). Final BCVA analysis by sub-groups demonstrated that 35 (81.4%) eyes remained stable, five (11.6%) eyes improved two or more ETDRS lines of BCVA, and three (7%) eyes decreased two or more ETDRS lines of BCVA. These visual improvements were accompanied by a statistically significant decrease in OCT CMT [23-24]. 

 Of the 43 eyes, 17 (39.5%) eyes showed total regression of retinal neovascularization. Fifteen (34.9%) eyes demonstrated partial regression of retinal neovascularization, and 11 (25.6) eyes showed no regression of retinal neovascularization. When divided by type of retinal neovascularization, of the total of 43 eyes, 15 (34.9%) eyes showed total regression of NVE, 10 (23.3%) eyes demonstrated partial regression of NVE, and 12 (27.9%) eyes showed no regression of NVE. Thirteen (30.2%) eyes showed total regression of NVD, 14 (32.5%) eyes demonstrated partial regression of NVD, and 16 (37.2%) eyes showed no regression of NVD [24]. Interestingly, the above 24 month neovascularization regression data compares unfavorably with our previously published six-month data [26]. This suggests that anti-VEGF treatment of PDR is not a permanent solution and that consolidation with either PRP and vitrectomy is needed.

## CONCLUSION

Given the off-label cost effective nature of intravitreal bevacizumab, its clinical effectiveness and safety have not been as thoroughly studied as other approved similar drugs. Our results may add evidence that IVB should be used as an adjuvant to PRP in patients with PDR, and that PRP and/or vitrectomy is necessary to control PDR in the long-run in a larger number of patients. 
